# Supplementary material for: Discovery of active mouse, plant and fungal cytochrome P450s in endogenous proteomes and upon expression in planta
Source: Sci Rep. 2024 May 2;14:10091. doi: 10.1038/s41598-024-60333-x (PMC11066006; doi:10.1038/s41598-024-60333-x)
Supplement: Supplementary file 8 — Supplementary Information 8. [file 41598_2024_60333_MOESM8_ESM.pdf]

Supplementary Methods, Figures and Tables

Font Farre *et al.* 'Discovery of active mouse, plant and fungal cytochrome P450s in endogenous proteomes and upon expression *in planta*.'

Supplementary Methods Synthesis of CYP450 probes

1. Synthesis of methyl 1-bromo-2-naphthoate

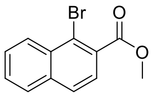

According to the modified procedure of Krätzschmar *et al.*:[2] sulfuric acid (5 drops) was added to a stirred suspension of 1-bromo-2-naphthoic acid (1.0 g, 4.0 mmol, 1.0 equiv.) and MeOH (10 mL) and the reaction mixture was heated under reflux for 18 h. The reaction mixture was cooled to rt and then solvent was removed *in vacuo* before being redissolved in EtOAc (15 mL). The organic layer was washed with sat. $NaHCO_3$ solution (20 mL) and H_2O (2 × 20 mL), dried ($MgSO_4$), filtered, and the solvent removed *in vacuo* to give the title compound as a colourless oil (1.1 g, 3.9 mmol, 97%) which was used without further purification; R_f 0.40 (20% EtOAc/ petroleum ether 40 – 60); ^{1}H NMR (400 MHz, $CDCl_3$) δ 8.46 (ddt, *J* = 8.5, 1.5, 1.0 Hz, 1H, ArC**H**), 7.88 – 7.81 (m, 2H, 2 × ArC**H**), 7.71 – 7.57 (m, 3H, 3 × ArC**H**), 4.01 (s, 3H, C**H**$_3$); ^{13}C NMR (101 MHz, $CDCl_3$) δ 167.9 (**C**=O), 135.2 (**C**CO_2Me), 132.3 (Ar**C**), 131.3 (Ar**C**), 128.6 (Ar**C**H), 128.2 (Ar**C**H), 128.1 (Ar**C**H), 127.8 (Ar**C**H), 125.8 (Ar**C**H), 122.6 (Ar**C**H), 52.7 (**C**H_3). One aromatic carbon resonance is unresolved. Data are in accordance with the literature.[2]

2. Synthesis of methyl 1-((trimethylsilyl)ethynyl)-2-naphthoate

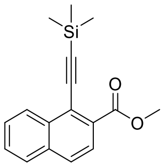

According to the modified procedure of Wright *et al.*:[1] bis(triphenylphosphine)palladium(II) chloride (0.04 g, 0.096 mmol, 2.0 mol%) was added to a solution of methyl 1-bromo-2-naphthoate (0.50 g, 1.9 mmol, 1.0 equiv.), ethynyltrimethylsilane (0.55 mL, 3.8 mmol, 2.0 equiv.), triethylamine (0.51 mL, 3.8 mmol, 2.0 equiv.), and copper(I) iodide (0.020 g, 0.23 mmol, 4.0 mol%) in MeCN (10 mL). The reaction mixture was heated to 90 °C and stirred for 16 h. The solvent was removed *in vacuo* and the resulting crude residue purified by flash column chromatography (1% EtOAc/petroleum ether 40-60 → 2% EtOAc/petroleum ether 40-60) yielding the title compound (0.19 g, 0.034 mmol, 35 %) as a brown solid. ^{1}H NMR (400 MHz, $CDCl_3$) δ 8.57 – 8.52 (m, 1H, ArC**H**), 7.92 (d, *J* 8.5 Hz, 1H, ArC**H**), 7.86 – 7.82 (m, 2H, ArC**H**), 7.63 – 7.61 (m, 2H, ArC**H**), 3.99 (s, 3H, OC**H**$_3$), 0.37 (s, 9H, Si(C**H**$_3$)$_3$); *m/z* (ES$^+$) 283.3 (M+H$^+$, 100%). Data are in accordance with the literature.[1]

3. Synthesis of 1-ethynyl-2-naphthoic acid

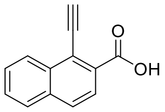

According to the modified procedure of Wright *et al.*:[1] a solution of 1 M NaOH (2 mL) was added dropwise to a stirred solution of methyl 1-((trimethylsilyl)ethynyl)-2-naphthoate (0.12 g, 0.42 mmol, 1.0 equiv.) in 2:1 EtOH:CH_2Cl_2 (4 mL) and then the reaction mixture was stirred at rt for 16 h. After this time, the reaction was quenched by dropwise addition of 2 M HCl (3 mL) until precipitate formed. The organics were extracted with EtOAc (3 × 8 mL), washed with brine (10 mL), dried ($MgSO_4$), filtered and the solvent removed *in vacuo* yielding the title compound as a brown solid (0.08 g, 0.41 mmol, 97%) which required no further purification. ^{1}H NMR (400 MHz, acetone-d_6) δ 8.51 – 8.46 (m, 1H, ArC**H**), 7.98 – 7.92 (m, 2H, 2 × ArC**H**), 7.89 (d, J = 8.5, Hz, 1H, ArC**H**), 7.65 – 7.60 (m, 2H, 2 × ArC**H**), 4.33 (s, 1H, CC**H**); *m/z* (ESI$^-$) 195 (M-H$^+$, 100%). Data are in accordance with the literature.[1]

4. Synthesis of 1-ethynyl-*N*-(hex-5-yn-1-yl)-2-naphthamide (DB089)

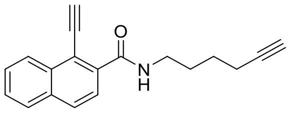

According to the modified procedure of Wright *et al.*:[1] propylphosphonic anhydride solution (≥50 wt. % in ethyl acetate, 0.12 mL, 0.36 mmol, 1.4 equiv.) was added to a stirred solution 1-ethynyl-2-naphthoic acid (0.050 g, 0.26 mmol, 1.0 equiv.), hex-5-yn-1-amine (0.040 g, 0.31 mmol, 1.2 equiv.), and DIPEA (0.18 mL, 1.0 mmol, 3.0 equiv.) in CH_2Cl_2 (6 mL) at room temperature. The reaction mixture was stirred at rt for 18 h. After this time, the solvent was removed *in vacuo* to give a crude residue which was further purified by column chromatography (20% EtOAc/ petrol 40-60 → 30% EtOAc/ petrol 40-60) yielding the title compound as a white solid (0.030 g, 0.052 mmol, 20%). ^{1}H NMR (400 MHz, $CDCl_3$) δ 8.45 (ddt, J = 8.5, 1.5, 1.0 Hz, 1H, ArC**H**), 7.99 – 7.83 (m, 3H, 3 × ArC**H**), 7.68 – 7.53 (m, 2H, 2 × ArC**H**), 7.21 (br t, J = 5.3 Hz, 1H, CON**H**), 3.93 (s, 1H, C**H**), 3.58 (td, J = 7.0, 5.5 Hz, 2H, C**H**$_2$), 2.29 (td, J = 7.0, 2.5 Hz, 2H, C**H**$_2$), 1.98 (t, J = 2.5 Hz, 1H, C**H**), 1.89 – 1.75 (m, 2H, C**H**$_2$), 1.74 – 1.67 (m, 2H, C**H**$_2$); ^{13}C NMR (101 MHz, $CDCl_3$) δ 167.1 (**C**=O), 136.4 (**C**CONH), 133.7 (Ar**C**), 133.3 (Ar**C**), 129.6 (Ar**C**H), 128.3 (Ar**C**H), 127.7 (Ar**C**H), 127.7 (Ar**C**H), 126.9 (Ar**C**H), 125.6 (Ar**C**H), 116.1 (**C**C≡CH), 89.1 (CC≡**C**H), 84.1 (C≡**C**H), 80.0 (C**C**≡CH), 68.8 (C≡**C**H), 39.7 (**C**H$_2$), 28.5 (**C**H$_2$), 25.9 (**C**H$_2$), 18.2 (**C**H$_2$); LRMS *m/z* (ES$^+$) 276.4 (M+H$^+$, 100%). Data are in accordance with the literature.[1]

6. Synthesis of methyl 6-((trimethylsilyl)ethynyl)-2-naphthoate

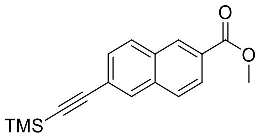

According to the modified procedure of Wright *et al.*:[1] bis(triphenylphosphine)palladium(II) chloride (0.040 g, 0.096 mmol, 5.0 mol%) was added to a solution of methyl 6-bromo-2-naphthoate (0.50 g, 1.9 mmol, 1.0 equiv.), ethynyltrimethylsilane (0.55 mL, 3.8 mmol, 2.0 equiv.), triethylamine (0.51 mL, 3.8 mmol, 2.0 equiv.), and CuI (0.020 g, 0.2 mmol, 10 mol%) in MeCN (10 mL). The reaction mixture was heated to 90 °C and stirred for 16 h. The reaction mixture was cooled to rt and the solvent was removed *in vacuo* to give a crude residue which was further purified by flash column chromatography (1% EtOAc/petroleum ether 40-60→ 2% EtOAc/petroleum ether 40-60) giving the title compound (0.51 g, 1.8 mmol, 94%) as an off white solid. ^{1}H NMR (400 MHz, $CDCl_3$) δ 8.57 – 8.55 (m, 1H, Ar**H**), 8.06 (dd, J = 8.5 1.5 Hz, 1H, Ar**H**), 8.03 – 8.00 (m, 1H, Ar**H**), 7.87 (d, J = 8.5 Hz, 1H, Ar**H**), 7.84 – 7.80 (m, 1H, Ar**H**), 7.56 (dd, J = 8.5, 1.5 Hz, 1H, Ar**H**), 3.98 (s, 3H, C**H$_3$**), 0.29 (s, 9H, Si(C**H$_3$**)$_3$); ^{13}C NMR (101 MHz, $CDCl_3$) δ 167.1 (**C**=O), 135.0 (**C**CO_2Me), 132.0 (Ar**C**), 131.7 (Ar**C**H), 130.8 (Ar**C**H), 129.4 (Ar**C**H), 129.3 (Ar**C**H), 128.1 (Ar**C**), 128.0 (Ar**C**H), 126.0 (Ar**C**H), 123.0 (**C**C≡C), 104.9 (**C**≡CSi), 96.3 (C≡**C**Si), 52.4 (**C**H$_3$), 0.0 (Si(**C**H$_3$)$_3$); LRMS *m/z* (ESI$^+$) 283 (M+H$^+$, 100%). Data are in accordance with the literature.[3]

7. Synthesis of 6-ethynyl-2-naphthoic acid

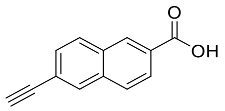

According to the procedure of Wright *et al.*:[1] a solution of 1 M NaOH (4 mL) was added dropwise to a stirred solution of methyl 6-((trimethylsilyl)ethynyl)-2-naphthoate (0.20 g, 0.71 mmol, 1.0 equiv.) in 2:1 EtOH:CH_2Cl_2 (8 mL). The reaction mixture was stirred at rt for 16 h before quenching by dropwise addition of HCl (6 mL) until the title compound precipitates. The aqueous layer was extracted with EtOAc (3 × 15 mL) and the organic layers combined, washed with brine (20 mL), dried ($MgSO_4$), filtered and the solvent removed *in vacuo* yielding the title compound as an off white solid (0.12 g, 0.68 mmol, 96%) which required no further purification. ^{1}H NMR (400 MHz, acetone-d_6) δ 8.67 (dt, J = 1.5, 0.5 Hz, 1H, ArC**H**), 8.18 (d, J = 1.5 Hz, 1H, ArC**H**), 8.17 – 8.10 (m, 2H, 2 × ArC**H**), 8.04 (dq, J = 8.5, 0.5 Hz, 1H, ArC**H**), 7.65 (dd, J = 8.5, 1.5 Hz, 1H, ArC**H**), 3.86 (s, 1H, CC**H**); LRMS *m/z* (ESI$^-$) 195 (M-H$^+$, 100%). Data are in accordance with the literature.[3]

8. Synthesis of 6-ethynyl-*N*-(hex-5-yn-1-yl)-2-naphthamide (DB096)

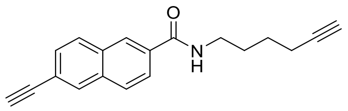

According to the modified procedure of Wright *et al.*:[1] propylphosphonic anhydride solution (≥50 wt. % in ethyl acetate, 0.23 mL, 0.77 mmol, 1.3 equiv.) was added to a stirred solution 6-ethynyl-2-naphthoic acid (0.10 g, 0.51 mmol, 1.0 equiv.), hex-5-yn-1-amine (0.080 g, 0.61 mmol, 1.1 equiv.), and DIPEA (0.25 mL, 1.5 mmol, 3.0 equiv.) in CH_2Cl_2 (6 mL) at room temperature. The reaction mixture

was stirred for 18 h at rt. After this time the solvent was removed *in vacuo* to give a crude residue which was further purified by flash column chromatography (20% EtOAc/petroleum ether 40-60 → 30% EtOAc/petroleum ether 40-60) to give the title compound (0.060 g, 0.22 mmol, 43%) as a white solid. ^{1}H NMR (500 MHz, $CDCl_3$) δ 8.23 (d, *J* = 1.5 Hz, 1H, Ar**H**), 8.02 (d, *J* = 1.5 Hz, 1H, Ar**H**), 7.87 – 7.81 (m, 3H, 3 × Ar**H**), 7.56 (dd, *J* = 8.5, 1.5 Hz, 1H, Ar**H**), 6.41 (br t, *J* = 5.5 Hz, 1H, N**H**), 3.54 (td, *J* = 7.0, 5.5 Hz, 2H, NHC**H₂**), 3.20 (s, 1H, ArC≡C**H**), 2.27 (td, *J* = 7.0, 2.5 Hz, 2H, C**H₂**C), 1.98 (t, *J* = 2.5 Hz, 1H, CH_2C≡C**H**), 1.79 (tt, *J* = 7.5, 6.5 Hz, 2H, $NHCH_2C\mathbf{H_2}$), 1.70 – 1.60 (m, 2H, $C\mathbf{H_2}CH_2C$); ^{13}C NMR (126 MHz, $CDCl_3$) δ 167.3 (**C**=O), 134.1 (Ar**C**), 132.8 (Ar**C**), 132.2 (Ar**C**), 132.0 (Ar**C**H), 129.5 (Ar**C**H), 129 (Ar**C**H), 128.3 (Ar**C**H), 127.1 (Ar**C**H), 124.4 (Ar**C**H), 121.3 (Ar**C**), 84.1 (Ar**C**≡CH), 83.6 (CH_2**C**≡CH), 78.6 (ArC≡**C**H), 68.9 (CH_2C≡**C**H), 39.7 (NH**C**H_2), 28.7 $NHCH_2\mathbf{C}H_2$, 25.8 ($\mathbf{C}H_2CH_2C$), 18.2 ($\mathbf{C}H_2C$); LRMS *m/z* (ESI$^+$) 276.4 (M+H$^+$, 100%); HRMS *m/z* (ESI$^+$): found 276.1383, $C_{19}H_{18}ON$ (M+H$^+$) requires 276.1383. Data are in accordance with the literature.[1]

9. Synthesis of methyl 4-bromobenzoate

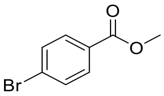

According to the procedure of Galán *et al.*:[4] Sulfuric acid (5 drops) was added to a stirred solution of 4-bromobenzoic acid (1.0 g, 5.0 mmol, 1.0 equiv.) in MeOH (13 mL) and the reaction mixture was heated to reflux for 18 h. After this time, the reaction mixture was cooled to rt and the solvent was removed *in vacuo*. The resulting crude residue was resuspended in EtOAc (15 mL) and washed with sat. $NaHCO_3$ (20 mL), H_2O (2 × 20 mL), dried ($MgSO_4$), filtered and then the solvent removed *in vacuo* to give the title compound (1.1 g, 5.0 mmol, 99%) as a white solid which required no further purification. ^{1}H NMR (400 MHz, $CDCl_3$) δ 7.86 – 7.79 (m, 2H, 2 × ArC**H**), 7.54 – 7.47 (m, 2H, 2 × ArC**H**), 3.84 (s, 3H, $CO_2C\mathbf{H_3}$); ^{13}C NMR (101 MHz, $CDCl_3$) δ 166.4 (**C**=O), 131.7 (Ar**C**H), 131.1 (Ar**C**H), 129.1 (Ar**C**), 128.0 (Ar**C**), 52.3 ($CO_2\mathbf{C}H_3$). Data are in accordance with the literature.[5]

10. Synthesis of methyl 4-((trimethylsilyl)ethynyl)benzoate

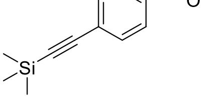

According to the modified procedure of Wright *et al.*: bis(triphenylphosphine)palladium(II) chloride (0.040 g, 0.096 mmol, 2.0 mol%) was added to a solution of methyl 4-bromobenzoate (0.41 g, 1.9 mmol, 1.0 equiv.), ethynyltrimethylsilane (0.55 mL, 3.8 mmol, 2.0 equiv.), triethylamine (0.51 mL, 3.8 mmol, 2.0 equiv.), and copper(I) iodide (0.020 g, 0.23 mmol, 4.0 mol%) in MeCN (10 mL). The reaction mixture was heated to 90 °C and stirred for 16 h. The solvent was removed *in vacuo* and the resulting crude residue purified by flash column chromatography (1% EtOAc/petroleum ether 40-60 → 2%

EtOAc/petroleum ether 40-60) giving the title compound (0.19 g, 0.034 mmol, 93 %) as an off-white solid. ^{1}H NMR (400 MHz, $CDCl_3$) δ 8.01 – 7.92 (m, 2H, 2 × Ar**H**), 7.55 – 7.47 (m, 2H, 2 × Ar**H**), 3.91 (s, 3H, CO_2C**H_3**), 0.26 (s, 9H, Si(C**H_3**)$_3$); ^{13}C NMR (101 MHz, $CDCl_3$) δ 166.4 (**C**O_2CH_3), 131.8 (Ar**C**H), 129.6 (Ar**C**), 129.3 (Ar**C**H), 127.7 (Ar**C**), 104.0 (**C**≡CSi), 97.6 (C≡**C**Si), 52.1 (CO_2**C**H_3), -0.2 Si(**C**H_3)$_3$; LRMS *m/z* (ESI$^+$) 233.1 (M+H$^+$, 100%) Data are in accordance with the literature.[6]

11. Synthesis of 4-ethynylbenzoic acid

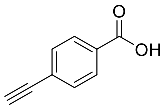

According to the modified procedure of Wright *et al*.:[1] lithium hydroxide hydrate (0.32 g, 7.5 mmol, 5.0 equiv.) was added to a stirred solution of 4-((trimethylsilyl)ethynyl)benzoic acid (0.35 g, 1.5 mmol, 1.0 equiv.) in THF (10 mL) and H_2O (5 mL) and the reaction mixture was vigorously stirred at rt for 18 h. After this time, the THF was removed *in vacuo* and the remaining aqueous layer was acidified by addition of pH 1 sulfate buffer. The aqueous phase was extracted with EtOAc (3 × 10 mL), dried ($MgSO_4$), filtered and the solvent removed *in vacuo* to give the title compound (0.22 g, 1.0 mmol, 67%) as a bronze coloured solid which required no further purification. ^{1}H NMR (400 MHz, $CDCl_3$) δ 7.85 (d, *J* = 6.5 Hz, 2H, 2 × Ar**H**), 7.47 – 7.36 (m, 2H, 2 × Ar**H**), 3.14 (s, 1H, C≡C**H**); ^{13}C NMR (101 MHz, $CDCl_3$) δ 167.7 (**C**=O), 131.8 (Ar**C**H), 131.0 (Ar**C**), 129.5 (Ar**C**H), 126.3 (Ar**C**), 82.8 (C≡CH), 80.0 (C≡CH); LRMS *m/z* (ESI$^-$) 145.1 (M-H$^+$, 100%). Data are in accordance with the literature.[7]

12. Synthesis of 4-ethynyl-*N*-(hex-5-yn-1-yl)benzamide (DB096)

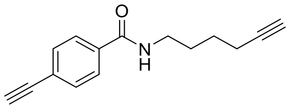

Propylphosphonic anhydride solution (50% in EtOAc, 0.25 mL, 1.0 mmol, 1.4 equiv.) was added to a stirred solution of 4-ethynylbenzoic acid (0.10 g, 0.68 mmol, 1.0 equiv.), hex-5-yn-1-amine hydrochloride (0.11 g, 0.82 mmol, 1.2 equiv.) and *N*,*N*-diisopropylethylamine (0.38 mL, 2.4 mmol, 3.0 equiv.) in CH_2Cl_2 (6 mL) and the reaction mixture was stirred for 1h. After this time, the solvent removed *in vacuo* and the resulting crude residue was purified by flash column chromatography (10% EtOAc/ petroleum ether 40-60 → 30% EtOAc/ petroleum ether 40-60) giving the title compound (0.096 g, 0.43 mmol, 63%) as a white solid. ^{1}H NMR (500 MHz, $CDCl_3$) δ 7.77 – 7.71 (m, 2H, 2 × ArC**H**), 7.58 – 7.52 (m, 2H, 2 × ArC**H**), 6.31 (d, *J* = 6.0 Hz, 1H, N**H**), 3.50 (td, *J* = 7.0, 5.5 Hz, 2H, NHC**H_2**), 3.21 (s, 1H, ArC≡C**H**), 2.28 (td, *J* = 7.0, 2.5 Hz, 2H, C**H_2**C), 2.00 (t, *J* = 2.5 Hz, 1H, CH_2C≡C**H**), 1.82 – 1.72 (m, 2H, $NHCH_2C$**H_2**), 1.69 – 1.59 (m, 2H, C**H_2**C); ^{13}C NMR (126 MHz, $CDCl_3$) δ 166.8 (**C**=O),

134.7 (Ar**C**), 132.3 (Ar**C**H), 126.9 (Ar**C**H), 125.3 (Ar**C**), 84.0 (CH2**C**), 82.8 (Ar**C**≡CH), 79.5 (ArC≡**C**H), 68.9 (CH_2C≡**C**H), 39.6 (NH**C**H_2), 28.6 (NHCH_2**C**H_2), 25.7 (**C**$H_2$$CH_2$C), 18.1 (**C**$H_2$C); LRMS *m/z* (ESI+) 248.0 (M+Na+, 100%); HRMS *m/z* (ESI+): found 226.1227, $C_{15}H_{16}ON$ ($M+H^+$) requires 226.1226. Data are in accordance with the literature.[1]

13. Synthesis of ethyl 7-hydroxy-2-oxo-*2H*-chromene-3-carboxylate

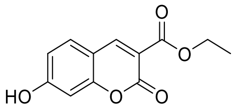

According to procedure of Vieira *et al.*:[8] piperidine (5 drops) was added to a stirred suspension of 2,4-dihydroxybenzaldehyde (0.10 g, 0.72 mmol, 1.0 equiv.) in diethyl malonate (0.23 g, 1.4 mmol, 2.0 equiv.) and the reaction mixture was stirred at rt for 18 h. The reaction mixture was acidified by addition of 2 M HCl (2 mL) until a precipitate formed. The solid was filtered and washed with Et_2O (3 × 10 mL) to give a crude residue that was further purified by flash column chromatography (70% EtOAc/CH_2Cl_2) giving the title compound as a pale yellow solid (0.090 g, 0.74 mmol, 53 %); ^{1}H NMR (400 MHz, DMSO-d_6) δ 11.08 (s, 1H, O**H**), 8.67 (s, 1H, C**H**CCO_2), 7.75 (d, *J* = 8.5 Hz, 1H, C**H**CCH), 6.84 (dd, *J* = 8.5, 2.5 Hz, 1H, CHC**H**COH), 6.72 (d, *J* = 2.0 Hz, 1H, HOCC**H**), 4.26 (q, *J* = 7.0 Hz, 2H, C**H_2**), 1.29 (t, *J* = 7.0 Hz, 3H, C**H_3**); LRMS *m/z* (ESI^+) 257.0 ($M+Na^+$, 100%). Data are in accordance with the literature.[8]

14. Synthesis of ethyl 2-oxo-7-(((trifluoromethyl)sulfonyl)oxy)-*2H*-chromene-3-carboxylate

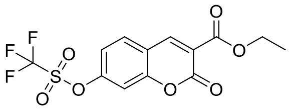

According to procedure of Starčević *et al.*:[9] triflic anhydride (0.74 mL, 4.2 mmol, 1.2 equiv.) was added dropwise to a stirred solution of ethyl 7-hydroxy-2-oxo-*2H*-chromene-3-carboxylate (0.83 g, 3.5 mmol, 1.0 equiv.) and pyridine (0.33 mL, 4.2 mmol, 1.2 equiv.) in CH_2Cl_2 (25 mL) cooled to 0 °C and the reaction mixture was stirred for 0.5 h. The reaction mixture was quenched by addition of H_2O (30 mL). The layers were separated and the aqueous layer was extracted with CH_2Cl_2 (3 × 10 mL), the organic layers were combined, dried ($MgSO_4$), filtered and solvent removed *in vacuo* to give a crude residue which was further purified by flash column chromatography (20% EtOAc/ petroleum ether 40-60) to give the title compound (0.52 g, 1.4 mmol, 41%) as a yellow solid; ^{1}H NMR (400 MHz, $CDCl_3$) δ 8.52 (s, 1H, C**H**CCO_2), 7.72 (d, *J* = 8.5 Hz, 1H, OCCHC**H**), 7.32 – 7.29 (m, 1H, OCC**H**CH), 7.28 (d, *J* = 6.0 Hz, 1H, OCC**H**), 4.43 (q, *J* = 7.0 Hz, 2H, C**H_2**), 1.42 (t, *J* = 7.0 Hz, 3H, CH_2); LRMS *m/z* (ESI^+) 367.3 ($M+H^+$, 100%). Data are in accordance with the literature.[9]

15. Synthesis of ethyl 2-oxo-7-((trimethylsilyl)ethynyl)-*2H*-chromene-3-carboxylate

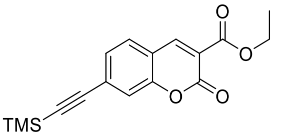

According to the modified procedure of Wright *et al.*:[1] bis(triphenylphosphine)palladium(II) chloride (0.038 g, 0.055 mmol, 5.0 mol%) was added to a solution of ethyl 2-oxo-7-(((trifluoromethyl)sulfonyl)oxy)-2*H*-chromene-3-carboxylate (0.40 g, 1.1 mmol, 1.0 equiv.), ethynyltrimethylsilane (0.31 mL, 2.2 mmol, 2.0 equiv.), triethylamine (0.29 mL, 2.2 mmol, 2.0 equiv.), and copper(I) iodide (0.023 g, 0.12 mmol, 10 mol%) in MeCN (10 mL) The reaction mixture was heated to 90 °C and stirred for 16 h. After this time, the solvent was removed *in vacuo* and the resulting crude residue purified by flash column chromatography (10% EtOAc/petroleum ether 40-60 → 20% EtOAc/petroleum ether 40-60) yielding the title compound (0.39 g, 1.0 mmol, 95%) as a yellow solid. ^{1}H NMR (400 MHz, $CDCl_3$) δ 8.47 (s, 1H, C**H**CCO_2), 7.52 (d, J = 8.0 Hz, 1H, ArC**H**), 7.42 – 7.33 (m, 2H, 2 × ArC**H**), 4.41 (q, J = 7.0 Hz, 2H, C$\mathbf{H_2}$), 1.41 (t, J = 7.0 Hz, 3H, C$\mathbf{H_3}$), 0.28 (s, 9H, Si(C$\mathbf{H_3}$)$_3$); *m/z* (ESI$^+$) 315 (M+H$^+$, 100%). Data are in accordance with the literature.[1]

16. Synthesis of 7-ethynyl-2-oxo-*2H*-chromene-3-carboxylic acid

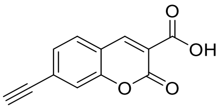

According to the modified procedure of Wright *et al.*:[1] a solution of 1 M NaOH (4 mL) was added dropwise to a stirred solution of ethyl 2-oxo-7-((trimethylsilyl)ethynyl)-2*H*-chromene-3-carboxylate (0.20 g, 0.63 mmol, 1.0 equiv.) in THF (8 mL). The reaction mixture was stirred at rt for 16 h. before quenching by dropwise addition of 2 M HCl (6 mL) to precipitate the title compound. The aqueous layer was extracted with EtOAc (3 × 15 mL) and the organic layers were combined, washed with brine (20 mL), dried ($MgSO_4$), filtered and the solvent removed *in vacuo* yielding the title compound as a yellow solid (0.10 g, 0.47 mmol, 75%) which required no further purification. ^{1}H NMR (400 MHz, CD_2Cl_2) δ 12.01 (s, 1H, COO**H**), 8.90 (d, J = 1.0 Hz, 1H, C**H**CCO_2), 7.75 (d, J = 8.0 Hz, 1H, ArC**H**), 7.59 (s, 1H, ArC**H**), 7.55 (dd, J = 8.0, 1.0 Hz, 1H, ArC**H**), 3.51 (s, 1H, CCHCHCC≡C**H**); *m/z* (ES$^-$) 213.3 (M-H$^+$, 100%). Data are in accordance with the literature.[1]

17. Synthesis of 7-ethynyl-*N*-(hex-5-yn-1-yl)-2-oxo-2*H*-chromene-3-carboxamide (DB086)

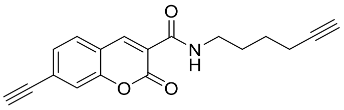

According to the modified procedure of Wright *et al.*:[1] propylphosphonic anhydride solution (≥50 wt. % in ethyl acetate, 0.10 mL, 0.35 mmol, 1.4 mmol) was added to a stirred solution of 7-ethynyl-2-oxo-2*H*-chromene-3-carboxylic acid (0.050 g, 0.23 mmol, 1.0 equiv.), hex-5-yn-1-amine (0.040 g, 0.28 mmol, 1.2 equiv.), and *N*,*N*-diisopropylethylamine (0.10 mL, 0.69 mmol, 3.0 equiv.) in CH_2Cl_2 (3 mL) and the reaction mixture was stirred at rt for 18 h. After this time, the solvent was removed *in vacuo* and the resulting crude residue further purified by flash column chromatography (20% EtOAc/ petroleum ether 40-60 → 30% EtOAc/ petroleum ether 40-60) giving the title compound as a white solid (0.050 g, 0.16 mmol, 70%). ^{1}H NMR (400 MHz, $CDCl_3$) 8.87 (d, *J* =0.5 Hz, 1H, C**H**CCO_2), 8.78 (br t, *J* = 5.8 Hz, 1H, CON**H**), 7.64 (d, *J* = 8.0 Hz, 1H, ArC**H**), 7.50-7.49 (m, 1H, ArC**H**), 7.46 (dd, *J* = 8.0 Hz, 1.5, ArC**H**), 3.49 (td, *J* = 7.0, 5.8 Hz, C**H**$_2$) 3.35 (s, 1H, C**H**), 2.26 (td, *J* = 7.0, 2.5 Hz, 2H, C**H**$_2$), 1.97 (t, *J* = 2.5 Hz, 1H, C**H**), 1.80 – 1.73 (m, 2H, C**H**$_2$), 1.66 – 1.60 (m, 2H, C**H**$_2$); LRMS *m/z* (ESI^+) 294.3 ($M+H^+$, 100%). Data are in accordance with the literature.[1]

18. Synthesis of 9-hydroxy-*7H*-furo[3,2-*g*]chromen-7-one

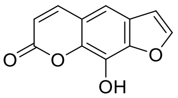

According to procedure of Shen *et al.*:[10] boron tribromide (0.19 mL, 2.0 mmol, 2.0 equiv.) was added to a vigorously stirred solution of 9-methoxy-*7H*-furo[3,2-*g*]chromen-7-one (0.22 g, 1.0 mmol, 1.0 equiv.) in CH_2Cl_2 (9 mL) at 0 °C, the reaction mixture was warmed to rt and stirred for 16 h. After this time, the reaction mixture was cooled to 0 °C and quenched by slow addition of saturated Na_2CO_3 (10 mL), the organics were extracted with EtOAc (3 × 15 mL). The organic layers were dried ($MgSO_4$), filtered, and the solvent removed *in vacuo* to give the title compound as a white solid (0.17 g, 0.85 mmol, 85%) which required no further purification. ^{1}H NMR (400 MHz, DMSO-d_6) δ 10.65 (1H, s, O**H**), 8.12 (1H, d, *J* = 9.5, C**H**), 8.07 (1H, dd, *J* = 2.0, 1.0, ArC**H**), 7.45 (1H, d, *J* = 1.5, ArC**H**), 7.04 (1H, dd, *J* = 2.0, 0.5, ArC**H**), 6.40 (1H, d, *J* = 9.5, C**H**).

19. Synthesis of methyl 2-((7-oxo-*7H*-furo[3,2-*g*]chromen-9-yl)oxy)acetate

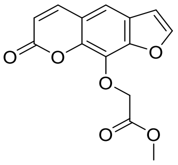

According to procedure of Wright *et al.*:[1] bromomethyl acetate (0.10 mL, 1.02 mmol, 1.2 equiv.) was added dropwise to a stirred solution of 9-hydroxy-*7H*-furo[3,2-*g*]chromen-7-one (0.17 g, 0.85 mmol, 1.0 equiv.) and potassium carbonate (0.35 g, 2.55 mmol, 3.0 equiv.) in DMF (5 mL), the reaction mixture was stirred at rt for 16 h. The solvent was removed under a stream of N_2 and the resulting crude residue was redissolved in EtOAc (10 mL) and washed with H_2O (3 × 20 mL). The organic phase was dried ($MgSO_4$), filtered, and solvent removed *in vacuo* yielding the title compound as a white solid

(0.20 g, 0.73 mmol, 86%) which required no further purification. ^{1}H NMR (500 MHz, $CDCl_3$) δ 7.79 (d, *J* = 9.5 Hz, 1H, O=CC**H**), 7.71 (d, *J* = 2.0 Hz, 1H, ArC**H**), 7.41 (s, 1H, ArC**H**), 6.84 (d, *J* = 2.0 Hz, 1H, ArC**H**), 6.40 (d, *J* = 9.5 Hz, 1H, C**H**), 5.17 (s, 2H, C**H**$_2$), 3.82 (s, 3H, C**H**$_3$); ^{13}C NMR (126 MHz, $CDCl_3$) δ 169.3 (**C**=O), 160.1 (**C**=O), 147.2 (Ar**C**), 146.8 (Ar**C**H), 144.3 (**C**H), 142.7 (Ar**C**), 130.8 (Ar**C**), 126.1 (Ar**C**), 116.6 (Ar**C**), 114.9 (**C**H), 113.6 (Ar**C**H), 106.8 (Ar**C**H), 69.0 (**C**H$_2$), 52.3 (**C**H$_3$); LRMS *m/z* (ESI$^+$) 275 (M+H$^+$, 100%); HRMS *m/z* (ESI$^+$): found 275.0550, $C_{14}H_{11}O_6$ (M+H$^+$) requires 275.0550. Data are in accordance with the literature.[1]

20. Synthesis of 2-((7-oxo-*7H*-furo[3,2-g]chromen-9-yl)oxy)acetic acid

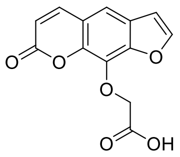

According to procedure of Wright *et al.*:[1] Lithium hydroxide (1 M in H_2O, 1.2 mL, 1.2 mmol, 2.0 equiv.) was added to a solution of methyl 2-((7-oxo-*7H*-furo[3,2-*g*]chromen-9-yl)oxy)acetate (0.17 g, 0.63 mmol) in THF (3 mL) and the reaction mixture stirred at rt for 16 h. After this time, the THF was removed *in vacuo* and the aqueous phase acidified with pH 1 sulfate buffer (5 mL). The organics were extracted with EtOAc (3 × 20 mL) and washed with brine (30 mL), dried ($MgSO_4$), filtered, and solvent removed *in vacuo* giving the title compound as an off white solid (0.14 g, 0.54 mmol, 85%) which required no further purification. ^{1}H NMR (400 MHz, DMSO-*d*$_6$) 8.16 (d, *J* = 9.5 Hz, 1H, C**H**), 8.12 (d, *J* = 2.0 Hz, 1H, ArC**H**), 7.66 (s, 1H, ArC**H**), 7.10 (d, *J* = 2.0 Hz, 1H, ArC**H**), 6.46 (d, *J* = 9.5 Hz, 1H, C**H**), 5.13 (s, 2H, C**H**$_2$); LRMS *m/z* (ESI$^-$) 259 (M−H$^+$, 100%). Data are in accordance with the literature.[1]

21. Synthesis of N-(Hex-5-yn-1-yl)-2-((7-oxo-*7H*-furo[3,2-*g*]chromen-9-yl)oxy)acetamide (DB080)

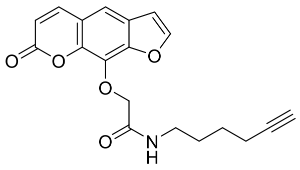

Propylphosphonic anhydride solution (≥50 wt. % in ethyl acetate, 0.15 mL, 0.52 mmol, 1.4 equiv.) was added to a stirred solution of 2-((7-oxo-*7H*-furo[3,2-*g*]chromen-9-yl)oxy)acetic acid **2.24** (0.10 g, 0.38 mmol, 1.0 equiv.), hex-5-yn-1-amine (0.050 g, 0.46 mmol, 1.2 equiv.), and DIPEA (0.50 mL, 1.0 mmol, 3.0 equiv.) in CH_2Cl_2 (6 mL) at room temperature. The reaction mixture was stirred overnight at rt. After this time, the reaction mixture was concentrated *in vacuo* to give a crude residue which was further purified by flash column chromatography (1% MeOH/CH_2Cl_2 → 2.5% MeOH/CH_2Cl_2) to give the title compound as a white solid (0.08 g, 0.25 mmol, 65%). ^{1}H NMR (400 MHz, Acetone-*d*$_6$) δ 8.05 (d, *J* = 10.0 Hz, 1H, OCOCHC**H**), 7.98 (d, *J* = 2.0 Hz, 1H, OCHC**H**), 7.66 (s, 1H, ArC**H**), 7.64 (br, 1H, N**H**), 7.03 (d, *J* =

2.0 Hz, 1H, OC**H**CH), 6.38 (d, *J* = 10.0 Hz, 1H, OCOC**H**CH), 4.87 (s, 2H, OC**H$_2$**), 3.37 (q, *J* = 7.0 Hz, 2H, NHC**H$_2$**), 2.31 (t, *J* = 2.5 Hz, 1H, C**H**), 2.21 (td, *J* = 7.0, 2.5 Hz, 2H, C**H$_2$**C), 1.75 – 1.63 (m, 2H, NHCH$_2$C**H$_2$**), 1.61 – 1.49 (m, 2H, C**H$_2$**CH$_2$C); ^{13}C NMR (101 MHz, Acetone-d_6) δ 168.3 (**C**=O), 160.2 (**C**=O), 148.5 (OCH**C**H), 148.3 (Ar**C**O), 145.6 (OCOCH**C**H), 144.1 (Ar**C**), 131.7 (Ar**C**), 127.2 (Ar**C**), 117.7 (Ar**C**), 115.7 (Ar**C**H), 115.4 (OCO**C**HCH), 108.0 (O**C**HCH), 84.8 (**C**≡CH), 73.1 (O**C**H$_2$), 70.2 (C≡C**H**), 39.0 (NH**C**H$_2$), 29.5 (NHCH$_2$**C**H$_2$), 26.6 (**C**H$_2$CH$_2$C), 18.5 (**C**H$_2$C); LRMS *m/z* (ESI$^+$) 340.2 (M+H$^+$, 100%). Data are in accordance with the literature.[1]

References

(1) Wright AT, Song JD, Cravatt BF. (2009) A suite of activity-based probes for human cytochrome P450 enzymes. *J. Am. Chem. Soc.* **131**, 10692–10700.

(2) Krätzschmar F, Kaßel M, Delony D, Breder A. (2015) Selenium-catalyzed C(Sp3)-H acyloxylation: application in the expedient synthesis of isobenzofuranones. *Chem. - A Eur. J.* **21**, 7030–7034.

(3) Klaasen H, Liu L, Meng X, Held PA, Gao HY, Barton D, Mück-Lichtenfeld C, Neugebauer J, Fuchs H, Studer A. (2018) Reaction selectivity in on-surface chemistry by surface coverage control—alkyne dimerization versus alkyne trimerization. *Chem. - A Eur. J.* **24**, 15303–15308.

(4) Galán LA, Cordes DB, Slawin AMZ, Jacquemin D, Ogden MI, Massi M, Zysman-Colman E. (2019) Analyzing the relation between structure and aggregation induced emission (AIE) properties of iridium(III) complexes through modification of non-chromophoric ancillary ligands. *Eur. J. Inorg. Chem.* **2019**, 152–163.

(5) Yu H, Wang J, Wu Z, Zhao Q, Dan D, Han S, Tang J, Wei Y. (2019) Aldehydes as potential acylating reagents for oxidative esterification by inorganic ligand-Supported iron catalysis. *Green Chem.* **21**, 4550–4554.

(6) Sharber, SA, Baral RN, Frausto F, Haas TE, Müller P, Thomas SW. (2017) Substituent effects that control conjugated oligomer conformation through non-covalent interactions. *J. Am. Chem. Soc.* **139**, 5164–5174.

(7) Mandl FA, Kirsch VC, Ugur I, Kunold E, Vomacka J, Fetzer C, Schneider S, Richter K, Fuchs TM, Antes I, Sieber SA. (2016) Natural-product-inspired aminoepoxybenzoquinones kill members of the gram-negative pathogen Salmonella by attenuating cellular stress response. *Angew. Chemie - Int. Ed.* **55**, 14852–14857.

(8) Vieira LCC, Paixão MW, Corrêa AG. (2012) Green synthesis of novel chalcone and coumarin derivatives via suzuki coupling reaction. *Tetrahedron Lett.* **53**, 2715–2718.

(9) Starčević Š, Brožič P, Turk S, Cesar J, Lanišnik Rižner T, Gobec S. (2011) Synthesis and biological evaluation of (6- and 7-phenyl) coumarin derivatives as selective nonsteroidal inhibitors of 17β-hydroxysteroid dehydrogenase type 1. *J. Med. Chem.* **54**, 248–261.

(10) Shen, Z.; Zhang, S.; Geng, H.; Wang, J.; Zhang, X.; Zhou, A.; Yao, C.; Chen, X.; Wang, W. (2019) Trideuteromethylation enabled by a sulfoxonium metathesis reaction. *Org. Lett.* **21**, 448–452.

Supplementary Figure S1

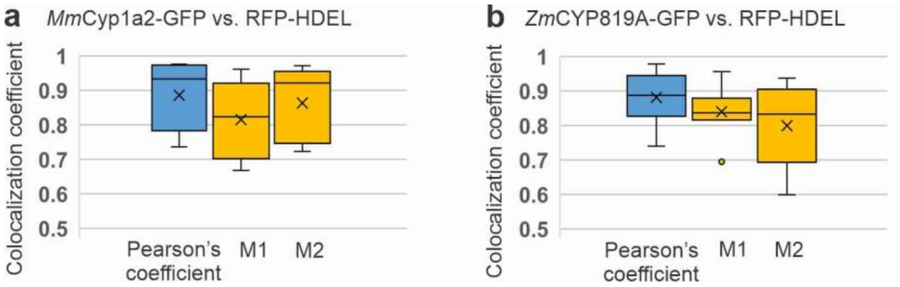

Figure S1 Fluorescence of P450-GFP and RFP-HDEL correlate in confocal microscopy. Colocalization of *Mm*Cyp1a2-GFP **(A)** or *Zm*CYP81A9-GFP **(B)** with RFP-HDEL was measured on a region of interest (ROI) on 10 images. Pearson's correlation coefficient (blue) and split Manders' correlation coefficients (M1 and M2, yellow) were calculated after Otsu thresholding. Shown are the mean values (x) with whiskers representing the maximum and minimum values for n=10 replicates. Signal correlation is considered strong when Pearson's coefficient value is high (above 0.8 to 1). Co-occurrence of RFP and GFP signal is tested with split Manders' coefficient M1 and M2, where M1 shows the fraction of pixels with RFP signals that overlaps with pixels with GFP signals, while M2 shows the fraction of pixels with GFP signal that overlaps with pixels with RFP signals above the threshold. M1 and M2 values range from 0 to 1, where 1 reflects perfect co-occurrence.

Uncropped gel images

Uncropped Figures 3c and 6b

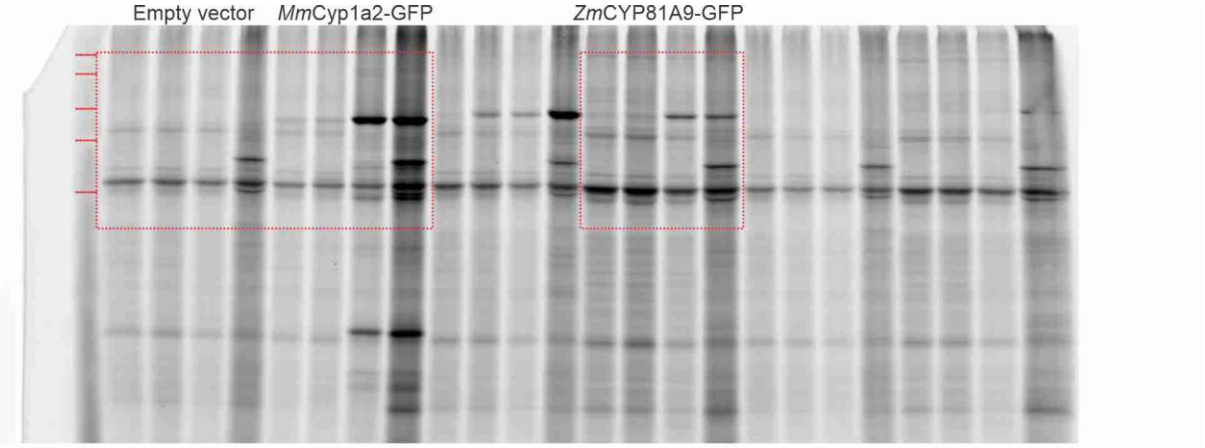

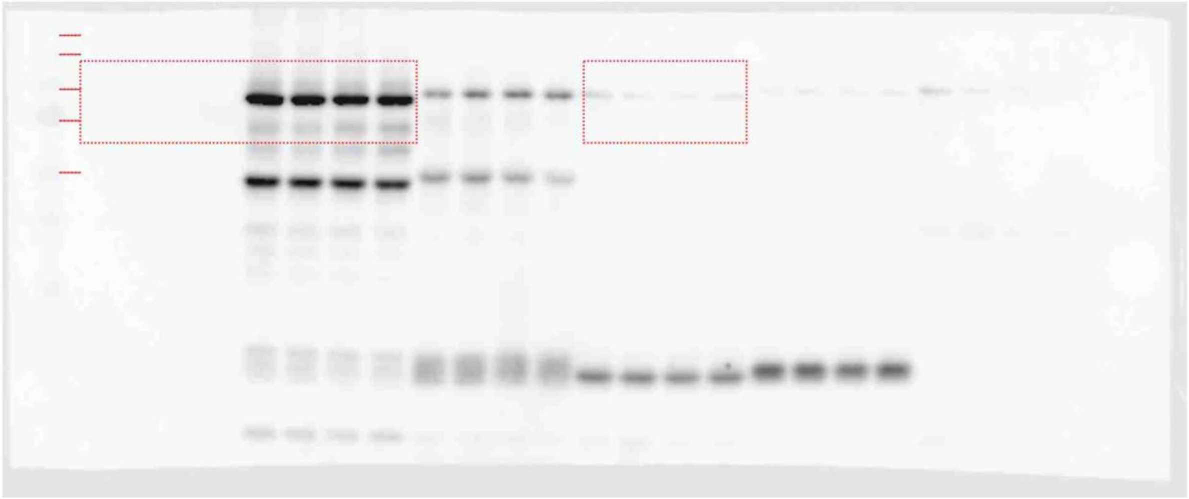

Uncropped Figure 5a

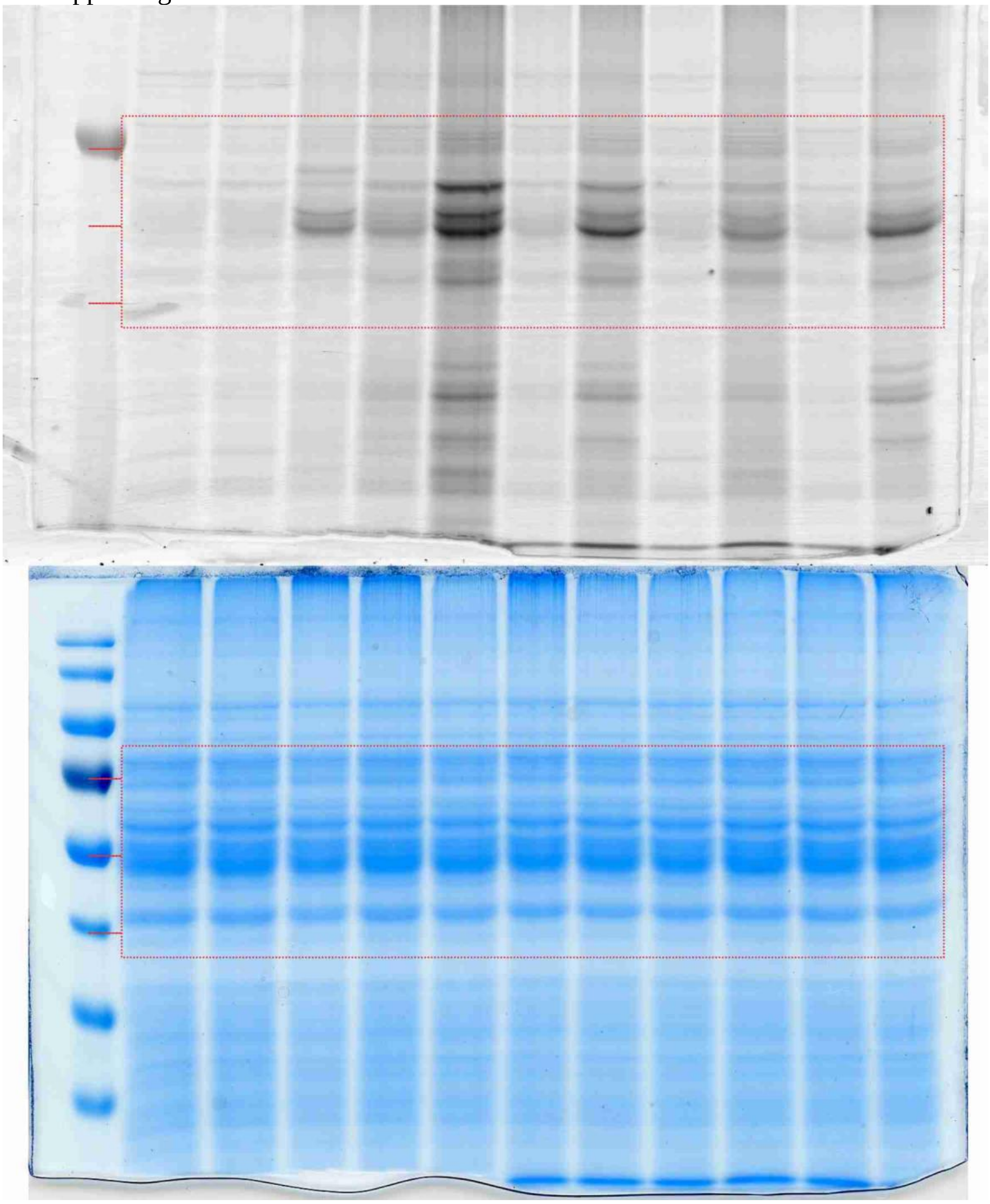

Uncropped Figure 8

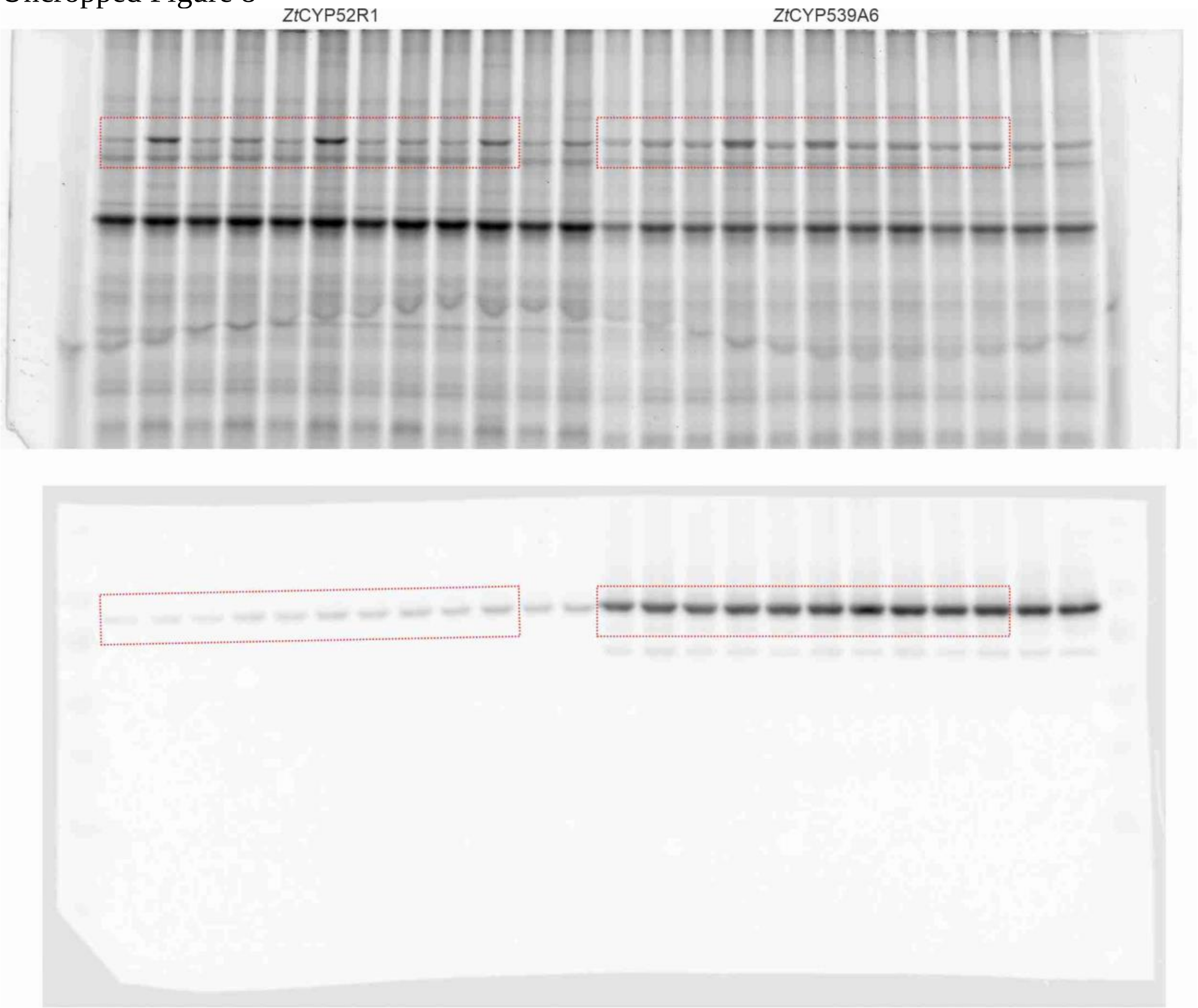

Uncropped Figure 8

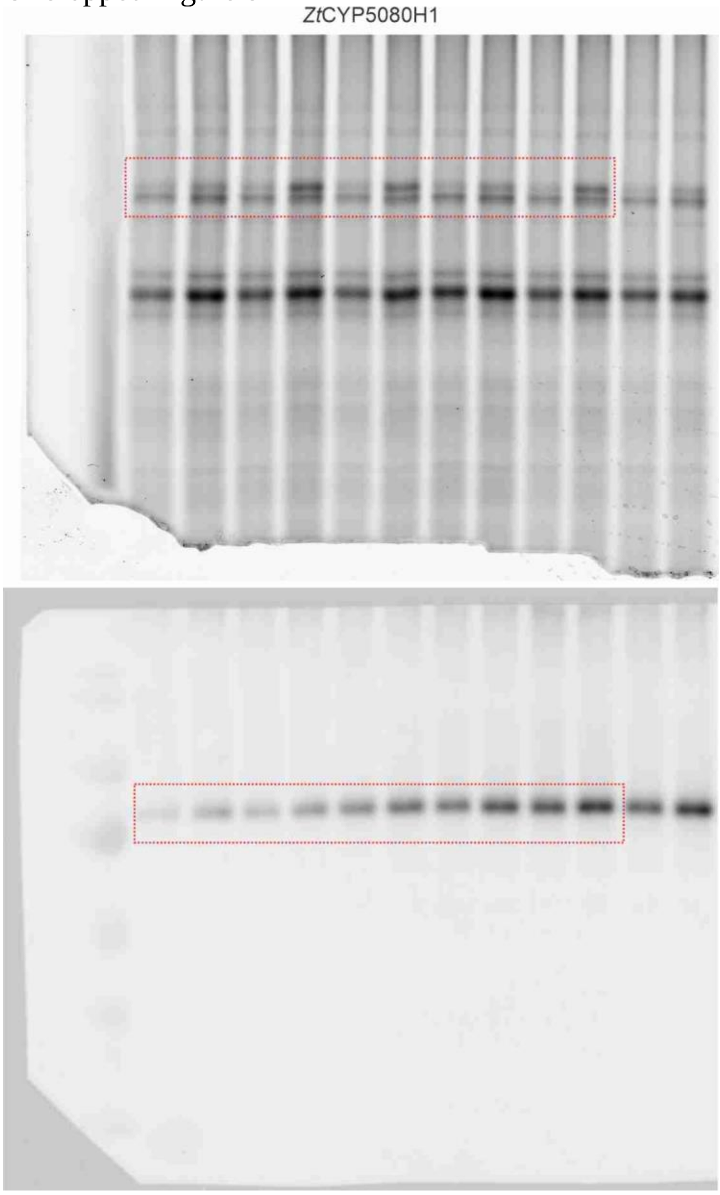

Uncropped Figure 8

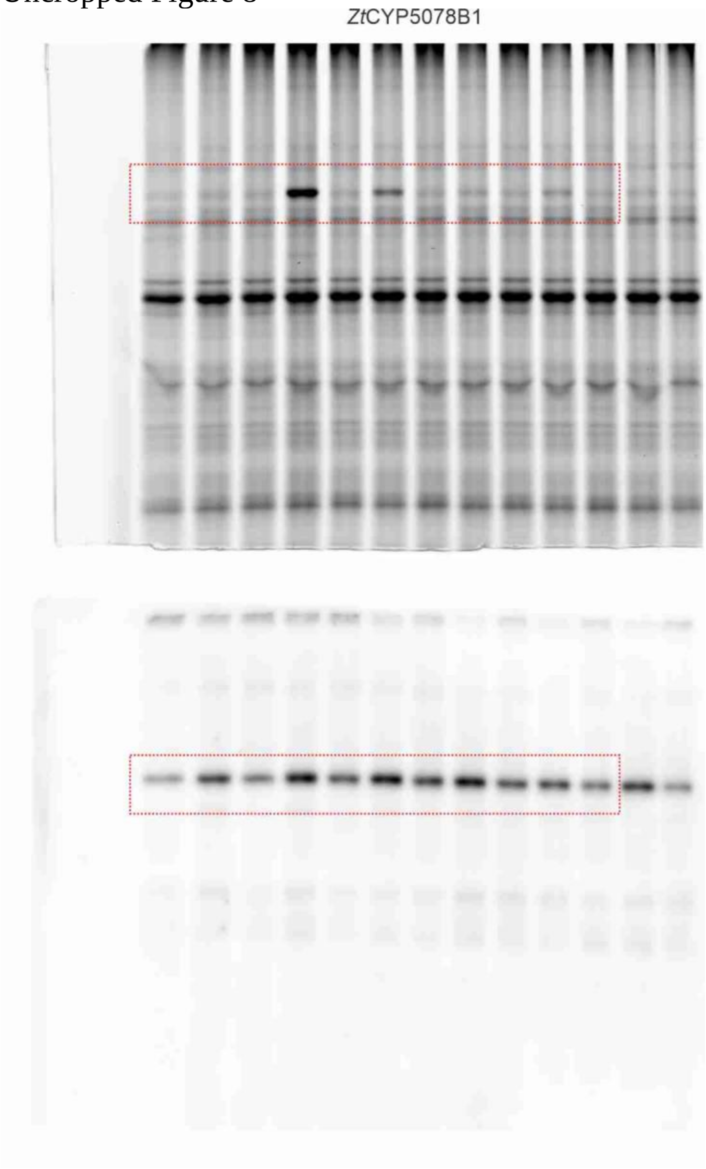

Supplementary Table S1 and S2

Font Farre *et al.* 'Discovery of active mouse, plant and fungal cytochrome P450s in endogenous proteomes and upon expression *in planta*.'

Table S1. Used plasmids

**Plasmid**	**Description**	**Reference**
pJK268c	pL1V2-P19-F2, Binary vector	Kourelis et al., 2020
GG1-01	pL0V-SC1-15456, Level 0 Cloning vector, EC15456.	Engler et al., 2014
GG1-55	pL0M-T-35S-1-41414, Level 0, 35S term., pICH41414	Engler et al., 2014
GG1-57	pL0M-PU-35S-TMV-3-51288, 2x35S prom. +TMVΩ, pICH51288.	Engler et al., 2014
GG1-78	pL0M-C2-eGFP-15095, Level 0 Module, eGFP for C terminal fusion. EC15095.	Engler et al., 2014
pMF115	pL2M-P19-kan-2x35S::*Mm*Cyp1a2-GFP	This work
pMF261	pL2M-P19-kan	This work
pMF325	pL2M-P19-kan-2x35S::*Zm*CYP81A9-GFP	This work
pMF330	pL2M-P19-kan-2x35S::*Zt*CYP5078B1	This work
pMF331	pL2M-P19-kan-2x35S::*Zt*CYP5080H1	This work
pMF348	pL2M-P19-kan-2x35S::*Zt*CYP52R1	This work
pMF349	pL2M-P19-kan-2x35S::*Zt*CYP539A6	This work

Table S2 Used oligonucleotides

**Oligo name**	**Sequence (5'-3')**
FW_ *Zt*CYP5078B1	TTGGTCTCAAATGGCTTTACCAGCACTTCTC
RV_ *Zt*CYP5078B1	TTGGTCTCACACCGCAAGATCGCTCTCTG
FW_ *Zt*CYP5080H1	TTGGTCTCAAATGTCGCTACTGACGG
RV_ *Zt*CYP5080H1	TTGGTCTCACACCTACCCTTGGGCTCAC
FW1_ *Zt*CYP52R1	TTGGTCTCAAATGCATAACGTCGCTCTG
FW2_ *Zt*CYP52R1	TTGGTCTCAGAAACCGGTATCGATTATTCTG
RV1_ *Zt*CYP52R1	TTGGTCTCATTTCCGCAGGGATGAAGATAG
RV2_ *Zt*CYP52R1	TTGGTCTCACACCCTCCGCCTCGTGCAAC
FW1_ *Zt*CYP539A6	TTGGTCTCAAATGCTCGTCGCCCTTATC
FW2_ *Zt*CYP539A6	TTGGTCTCAGAAACCCTCCGGCTTTACC
RV1_ *Zt*CYP539A6	TTGGTCTCATTTCATTCATTGTATGTGTGAGG
RV2_ *Zt*CYP539A6	TTGGTCTCACACCTGACCTGCTTGCCTTC

*Zm*CYP81A9

TTGAAGACAAAATGGACAAGGCTTACATTGCTGCTCTGTCTGCTGCTGCTCTTTTCCTTCTTCACTACCTGCTTGGTAGACGTGCTGGTGGTGAAGGTAAGGCTAAAGCTA
AGGGCTCTAGAAGAAGGCTGCCTCCTTCACCTCCTGCTATTCCTTTCTTGGGTCACCTCCATCTTGTGAAGGCTCCATTCCATGGTGCTCTTGCTAGACTTGCTGCTAGGCA
TGGTCCTGTGTTCTCTATGAGACTTGGAACTCGGAGGGCTGTCGTGGTTTCTTCACCTGATTGTGCTAGGGAATGCTTCACCGAGCACGATGTGAATTTCGCTAACAGGCC
TCTGTTCCCGTCTATGAGGCTTGCTTCTTTCGATGGCGCTATGCTGAGCGTGTCAAGCTATGGTCCTTATTGGAGGAACCTTAGGCGTGTTGCTGCTGTGCAACTTCTTTCA
GCTCATAGAGTGGGTTGCATGGCTCCAGCTATTGAAGCTCAGGTGAGAGCTATGGTGAGAAGGATGGATAGAGCTGCTGCAGCTGGTGGCGGTGGTGTTGCTAGAGTTC
AACTTAAGAGAAGGCTGTTCGAGCTGAGCCTGTCTGTGCTGATGGAAACTATTGCTCACACCAAGACCAGCAGAGCTGAGGCTGATGCTGATAGCGATATGTCTACTGA
GGCCCACGAGTTCAAGCAGATTGTGGATGAGCTTGTGCCTTACATCGGCACTGCTAACAGGTGGGATTACCTTCCTGTGCTGAGATGGTTCGATGTGTTCGGTGTGAGGA

ACAAGATCCTGGATGCTGTTGGTAGAAGGGATGCTTTCCTTGGCAGGCTTATCGATGGTGAGAGAAGGCGTCTTGATGCTGGTGATGAGAGCGAGAGCAAGTCAATGAT
TGCTGTGCTTCTGACCCTGCAGAAGTCTGAGCCTGAGGTTTACACCGATACCGTGATTACTGCTCTGTGCGCTAACCTTTTCGGTGCTGGTACTGAGACTACTTCTACTAC
TACCGAGTGGGCCATGAGCCTGCTTTTGAATCATAGAGAGGCACTGAAGAAGGCCCAGGCTGAAATTGATGCTGCTGTTGGAACATCTAGGCTGGTGACTGCTGATGAT
GTGCCTCACCTTACTTACCTGCAGTGCATCGTGGATGAGACTCTTAGGCTTCATCCTGCTGCTCCTCTGCTTTTGCCTCATGAATCTGCAGCTGATTGCACCGTTGGTGGTT
ACGATGTTCCTAGGGGTACTATGCTGCTTGTGAATGTGCATGCTGTGCATAGGGATCCTGCTGTGTGGGAAGATCCTGATAGATTCGTTCCCGAGAGGTTCGAAGGTGCT
GGCGGTAAGGCTGAAGGTAGACTTCTTATGCCTTTCGGCATGGGCAGACGTAAGTGTCCTGGTGAAACTCTTGCTCTTAGGACCGTGGGTCTTGTGCTTGCTACTTTGCTT
CAGTGCTTCGATTGGGATACCGTGGATGGTGCTCAAGTGGACATGAAGGCTTCAGGTGGTCTTACTATGCCTAGGGCTGTTCCTCTTGAGGCTATGTGCAGACCTAGAAC
TGCTATGAGGGGTGTGCTTAAGAGGCTGGGTGTTGTCTTCAA

*Mm*Cyp1a2

TTGAAGACAAAATGGCGTTCTCCCAGTACATCTCCTTAGCCCCAGAGCTGCTACTGGCCACTGCCATCTTCTGTTTAGTGTTCTGGATGGTCAGAGCCTCAAGGACCCAGG
TTCCCAAAGGCCTGAAGAATCCACCCGGACCCTGGGGCTTGCCCTTCATTGGGCACATGCTGACTGTGGGGAAGAACCCACACCTGTCACTGACACGGCTGAGTCAGCA
GTATGGGGACGTGCTGCAGATCCGCATCGGCTCCACTCCTGTGGTGGTGCTGAGCGGCCTGAACACCATCAAGCAGGCCCTGGTGAGGCAGGGAGATGACTTCAAGGGC
CGACCAGACCTCTACAGCTTCACACTTATCACTAACGGCAAGAGCATGACTTTCAACCCAGACTCTGGACCCGTGTGGGCTGCCCGCCGGCGCCTGGCCCAGGATGCCCT
GAAGAGCTTCTCCATAGCCTCGGACCCGACGTCAGCATCCTCTTGCTATTTGGAGGAGCACGTGAGCAAGGAGGCTAACCATCTCGTCAGCAAGCTTCAGAAGGCGATG
GCAGAGGTTGGCCACTTCGAACCAGTCAGCCAGGTGGTGGAATCGGTGGCTAACGTCATTGGTGCCATGTGCTTTGGGAAGAACTTCCCCCGGAAGAGCGAGGAGATGC
TGAACATCGTGAATAACAGCAAGGACTTTGTGGAGAATGTCACCTCAGGGAATGCAGTGGACTTCTTCCCGGTCCTGCGCTACCTGCCCAACCCGGCCCTCAAGAGGTTT
AAGACCTTCAATGATAACTTCGTGCTGTTTCTGCAGAAAACTGTCCAGGAGCACTACCAAGACTTCAACAAGAACAGTATCCAAGACATCACAAGTGCCCTGTTCAAGCA
CAGCGAGAACTACAAAGACAATGGCGGACTCATCCCCGAGGAGAAGATTGTCAACATTGTCAATGACATCTTTGGAGCTGGCTTTGACACAGTCACCACAGCCATCACC
TGGAGCATTTTGCTACTTGTGACATGGCCTAACGTGCAGAGGAAGATCCATGAGGAGCTGGACACGGTGGTTGGCAGGGATCGGCAACCACGGCTTTCTGACCGTCCCC
AGCTGCCATATCTAGAGGCCTTCATCCTGGAGATCTACCGATACACATCCTTTGTCCCCTTCACCATCCCCCACAGCACAACGAGGGACACCTCACTGAATGGCTTCCAC
ATTCCCAAGGAGCGCTGTATCTACATAAACCAGTGGCAGGTCAACCATGATGAGAAGCAGTGGAAAGACCCCTTTGTGTTCCGCCCAGAGCGGTTTCTTACCAATAACA
ACTCGGCCATCGACAAGACCCAGAGCGAGAAGGTGATGCTCTTCGGCTTGGGAAAGCGCCGGTGCATTGGGGAGATCCCGGCCAAGTGGGAAGTATTCCTCTTCTTAGC
CATCCTGCTGCAGCATCTGGAGTTTAGTGTGCCACCGGGTGTGAAGGTGGACCTGACACCCAACTATGGGTTGACCATGAAGCCCGGGACCTGTGAACACGTCCAGGCA
TGGCCACGCTTTTCCAAGGGTGTTGTCTTCAA
